# Repetitive transcranial magnetic stimulation (rTMS) is associated with increased abstinence in substance use disorders and comorbid depression

**DOI:** 10.1192/j.eurpsy.2024.524

**Published:** 2024-08-27

**Authors:** S. El Hayek, H. Tolba, A. Elmougy, W. Foad

**Affiliations:** ^1^Erada Center for Treatment and Rehabilitation in Dubai, Dubai, United Arab Emirates

## Abstract

**Introduction:**

Substance use disorders (SUDs) are associated with high rates of comorbid depression. Finding effective treatments for many of the substances of abuse is still an area of developing research. Repetitive transcranial magnetic stimulation (rTMS) is an established treatment for depression, but its effects in SUDs are less conclusive.

**Objectives:**

Therefore, we aimed to investigate the effect of rTMS in patients with SUDs and comorbid major depressive disorder (MDD).

**Methods:**

We conducted a retrospective observational study of 55 patients with SUDs and comorbid MDD who were eligible for rTMS. Craving was measured using the Brief Substance Craving Scale (BSCS). Severity of MDD was measured using the Clinical Global Impression-Severity (CGI-S) scale.

**Results:**

We found a statistically significant difference between baseline and posttreatment scores in patients receiving rTMS on both CGI-S scores and BSCS scores. The number of rTMS sessions significantly predicted increased days of abstinence in the community, even after controlling for confounders.

**Conclusions:**

Patients with SUDs and MDD who received rTMS significantly improved in the areas of severity of depression and craving. The number of rTMS sessions significantly predicted increased abstinence.

**Disclosure of Interest:**

None Declared

